# The Impact of the COVID-19 Pandemic on Public Interest in the Energy Labelling on Restaurant Menus

**DOI:** 10.3390/nu15020466

**Published:** 2023-01-16

**Authors:** Areej A. Alkhaldy, Omar A. Alhumaidan, Sarah M. Alkhunein, Majid M. Alkhalaf, Khlood A. Bookari, Jamila M. Arrish

**Affiliations:** 1 Clinical Nutrition Department, Faculty of Applied Medical Sciences, King Abdulaziz University, P.O. Box 80215, Jeddah 21589, Saudi Arabia; 2 National Nutrition Committee (NNC), Saudi Food and Drug Authority (Saudi FDA), P.O. Box 13312, Riyadh 13513, Saudi Arabia; 3 Clinical Nutrition Department, Faculty of Applied Medical Sciences, Taibah University, P.O. Box 344, Medinah 42353, Saudi Arabia

**Keywords:** menu-labelling policy, nutrition, restaurants, interest

## Abstract

No study has investigated the effect of the COVID-19 pandemic on the public’s interest in using energy labelling on restaurant menus. This study explores the effects of the COVID-19 pandemic on the public interest in using energy labelling on restaurant menus and meal delivery applications and the impact of energy-labelling availability on food choices during the COVID-19 pandemic in Saudi Arabia. An online questionnaire was completed by 1657 participants aged ≥ 18 years. Before the COVID-19 pandemic, 32% of customers visited a restaurant 2–4 times/week. However, during the pandemic, 35% of customers visited a restaurant only once per week. There was no difference in interest in reading energy labelling or using meal delivery applications before and during the pandemic. During the COVID-19 pandemic, about 55% of restaurant customers reported that they had noticed energy labelling, with 42% of them being influenced by the energy-labelling information. Regarding energy information on food delivery applications, 40% of customers noticed energy labelling when using the applications, with 33% of them being affected by the energy labelling. Customer interest in reading about energy on restaurant menus during the pandemic did not change significantly from the level of interest before the pandemic. The interest expressed by the public in using the energy labelling was low both before and during the COVID-19 pandemic.

## 1. Introduction

The emergence of the COVID-19 global pandemic has added several challenges to human lives worldwide, causing remarkable impacts on health, lifestyle, social life, and the economy [1]. In addition, many national and international laws and regulations have been affected, including energy-labelling policies [2,3].

An example of one such impact is the U.S. Food and Drug Administration (FDA) energy-labelling policies. The FDA released a guidance document entitled Temporary Policy Regarding Nutrition Labelling of Standard Menu Items in Chain Restaurants and Similar Retail Food Establishments during the COVID-19 Public Health Emergency to provide temporary flexibility to restaurants and similar food retailers required to provide nutritional information, including calories, on menus and food delivery applications’ menu boards [3].

In Saudi Arabia, a menu calorie-labelling policy was announced in August 2018 by the Saudi Food and Drug Authority (SFDA). The policy required all restaurants, including dine-in restaurants, fast-food restaurants, cafes, ice cream shops, fresh-fruit juice shops, pastry shops, higher-educational institutions, and governmental agencies to post caloric information clearly in their menus by the end of December 2018 [4]. Several studies were conducted to evaluate public knowledge, attitudes, views, and practices towards the SFDA’s mandatory menu calorie-labelling policy and the impact of the policy on the public’s dietary behaviour. Overall, these studies reported adequate knowledge and positive attitudes and practices related to menu caloric labelling in Saudi Arabia. In addition, these studies highlighted the importance of menu caloric labelling as a valuable tool for promoting healthy eating habits and encouraging healthier food selection [5,6,7,8]. Unlike the US FDA, the SFDA did not release any temporary guidance to the restaurants in relation to displaying energy labelling during the COVID-19 pandemic.

Many studies have indicated that energy labelling may have a beneficial effect on food choices and is effective in reducing energy order and consumption [9]. During the COVID-19 pandemic, most studies have examined the effect of COVID-19 on eating habits, food intake, physical activities, and lifestyle [9,10,11,12,13,14,15,16,17,18,19], with no available data on the effect of the COVID-19 lockdown on public use and interest in using restaurant-menu caloric labelling worldwide, including in Saudi Arabia. Given the possibility of future public-health pandemics, it is essential to understand the interest of the public in using energy labelling during the COVID-19 public-health pandemic. A better understanding of public perception of energy labelling may provide important information that could be used to raise awareness on how to utilise energy labelling to improve food choices and reduce energy intake during a global health emergency.

It has been reported that negative weight-related behaviours, including unhealthy eating and physical inactivity during the COVID-19 pandemic, were associated with being overweight and obese, which could make these people more susceptible to chronic diseases [20]. To address obesity issues and high food consumption during the COVID-19 pandemic, it is recommended that the energy-labelling legislation be adopted to influence consumer eating choices and reduce the risk of obesity and its complications during any public health emergency.

This study aims to explore the effects of the COVID-19 pandemic on the public interest in using energy labels on restaurant menus and meal delivery applications and whether the availability of energy labelling during the COVID-19 pandemic impacted the public’s food choices. The findings of this study will help to understand the public’s response toward the caloric labelling of menus in health emergencies. Further, the data of this study could be used and put into consideration when developing the guidance document to instruct the restaurants so that the public can estimate their daily caloric intake and maintain a healthy lifestyle during any public health emergency.

## 2. Materials and Methods

### 2.1. Study Design

A cross-sectional questionnaire-based internet study was conducted from August 2020 to January 2021 by researchers from the National Nutrition Committee on the SFDA and researchers from King Abdulaziz University and Taibah University in the Kingdom of Saudi Arabia. The study was approved by the Biomedical Ethics Research Committee at King Abdulaziz University (Jeddah, Saudi Arabia) (Reference No. 469-20). Informed consent was obtained from all study participants.

### 2.2. Participants and Recruitment

A convenience sample of 1657 Saudi adult participants aged ≥ 18 was included. Participants were asked to complete a publicly distributed electronic questionnaire using Google Forms. The electronic questionnaire was distributed using the snowball-sampling method via various social media platforms, such as WhatsApp and Twitter. For WhatsApp, the link to the questionnaire was sent to all contacts in the personal contact lists of this study’s authors, including relatives, friends, and neighbors, who were encouraged to send the link to all their contacts within Saudi Arabia. In addition, the link was shared with many WhatsApp social groups. On Twitter, the account of the SFDA tweeted the survey link, and this study’s authors requested public figures with high numbers of followers in Saudi Arabia to re-tweet the study questionnaire.

### 2.3. Sample-Size Calculation

The sample size was calculated using an online calculator (Epi Info) [21] and data obtained from the General Authority for Statistics on 25,192,780 adult residents and citizens ≥ 18 years in the Kingdom of Saudi Arabia in 2019 [22]. The calculation showed that a sample of 1083 was adequate, with a 99.9% confidence interval, and hypothesised a 50% frequency-of-outcome factor in the population.

### 2.4. Study Questionnaire

The questionnaire was composed of three main sections, with 33 questions requiring approximately 15 min to answer. Four Ph.D. experts from nutritional and health backgrounds prepared the survey questions. The questionnaire was subjected to validity, reliability, and internal-consistency testing. Content validity was ascertained by 10 Ph.D. and M.Sc. experts in nutrition and dietetics, and a content validity index of 0.84 was achieved. The questionnaire was revised based on the experts’ feedback and comments. Test-retest reliability was carried out and expressed as a correlation coefficient with an average value of 0.79. Internal consistency was assessed by Cronbach’s alpha, and values for different subscales ranged between 0.72–0.88. The questionnaire was then revised based on their feedback and comments. The modifications included changing the response options, grouping and breaking down some questions, and including a few more questions. The questionnaire had five sections, as follows: (1) Demographic characteristics of the participants included age, sex, marital status, educational level, work status, household income, field of study, and geographic location. The participants’ background information included their perceived overall quality of diet, weight perception, whether they had been diagnosed with COVID-19, their medical status, smoking status, physical-activity level, and if they knew how to calculate the calories. In addition, participants were asked whether they agreed, disagreed, or were neutral about the usefulness of energy labelling during the COVID-19 pandemic. (2) Self-reported weight, body mass index (BMI), and quality of diet before and during the COVID-19 pandemic. (3) Frequency of restaurant visits and use of food delivery applications before and during the COVID-19 pandemic. (4) Interest in energy labelling in restaurant and food-delivery-application menus before and during the COVID-19 pandemic. (5) The public’s observation of the menu energy labelling and its impact on participants’ food choices during the COVID-19 pandemic.

### 2.5. Statistical Analysis

Data were checked and entered using standardised entry codes written on a Statistical Package for the Social Sciences (SPSS, Version 23.0; IBM Corp., Armonk, NY, USA) v26 data file. The data were presented as frequencies and proportions. Descriptive statistics were used to examine demographic characteristics and food frequency. To test the difference between two variables, a chi-squared test of independence was used with a *p*-value < 0.05, denoting statistical significance. Bonferroni correction was used due to multiple comparisons.

## 3. Results

### 3.1. Demographics and Background of Participants

Table 1 shows the general characteristics of the study participants (*n* = 1657). The most frequently reported characteristics of study participants were age between 18 and 24 years (52%), female (81%), single (58%), Saudi (93%), had a bachelor’s degree (53%), from a scientific background (38%), student (45%), approximately evenly drawn from across the income categories of no income (29%) or less than 2000 SR (29%), and from the west region (51%) (Table 1).

Regarding participant backgrounds, only 14% of the participants were diagnosed with COVID-19, 80% reported no diseases, 90% were non-smokers, and 58% were reasonably physically active. Approximately 40% knew how to calculate calories and 39% did not. Of the participants, 75% agreed that energy labelling during the COVID-19 pandemic was helpful.

### 3.2. Perceived Weight Status, Body Weight, and Quality of Diet

The majority (47%) of participants reported being within the normal weight range, and 31% reported that their weight was slightly above normal. However, during COVID-19, 33% of the participants reported that their weight had increased, and 32% reported that their weight had not changed. The reported BMI values showed that the participants’ BMI did not significantly change before and during the COVID-19 pandemic. Of the participants, 72% reported an adequate-to-healthy diet before the COVID-19 pandemic, with the majority (47%) reporting that the COVID-19 pandemic did not affect their diet. Only 21% of the participants reported that the COVID-19 pandemic worsened their diet (Table 2).

### 3.3. Restaurant Visits and Use of Food Delivery Applications before and during the COVID-19 Pandemic

**Figure 1:**
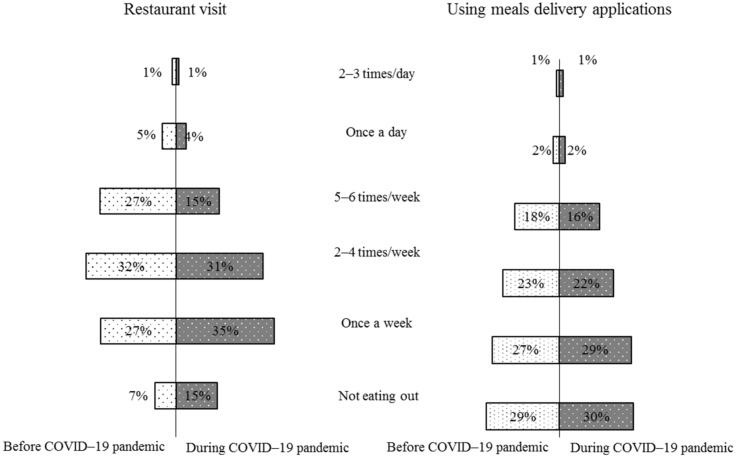
Frequency of restaurant visits and using food delivery applications before and after the COVID-19 pandemic (1657).

Before the COVID-19 pandemic, the percentage of restaurant visits (2–4 times/week) was the most frequent among the study participants (32%). However, during the COVID-19 pandemic, visiting restaurants once per week was the most frequent among the study participants (35%). In addition, there was an increase from 7% to 15% of the participants who reported not visiting a restaurant during the COVID-19 pandemic. However, there was no change in the use of food delivery applications before and during the COVID-19 pandemic (Figure 1).

### 3.4. Interest in Reading Energy Labelling before and during the COVID-19 Pandemic in Restaurants and Meal Delivery Applications

Table 3 shows participants’ interest in reading the energy labelling in restaurants and food delivery applications before and during the COVID-19 pandemic. The majority of participants were either somewhat interested (39%) or not interested (43%) in reading the energy labelling before the COVID-19 pandemic in restaurants. During the COVID-19 pandemic, the percentage of participants who were somewhat interested was 39%, with a slight decrease in the number of participants who were not interested at all (38%). Only 17% of the participants were very interested in reading the energy labelling in restaurants before the COVID-19 pandemic, and 22% were very interested during the COVID-19 pandemic. Age, marital status, educational level, and work status were significant variables associated with the interest in reading the energy labelling before and during the COVID-19 pandemic in restaurants (*p* < 0.05). In particular, single young females with bachelor’s degrees were seen to be more interested in reading the energy labelling during the COVID-19 pandemic.

Regarding the use of meal delivery applications, most participants were either somewhat interested (33%) or not interested (50%) in reading the energy labelling both before and during the COVID-19 pandemic. The percentage of participants who were somewhat interested was the same at 33%, with a slight decrease in the number of participants who were not interested at all (46%). Only 17% of the participants were extremely interested before the COVID-19 pandemic, and 20% were extremely interested during the COVID-19 pandemic. Age, marital status, educational level, and work status were significant variables associated with the interest in reading the energy labelling before and during the COVID-19 pandemic when using meal delivery applications (*p* < 0.05). In particular, single young participants with bachelor’s degrees were seen to be more interested in reading the energy labelling when using meal delivery applications during the COVID-19 pandemic. In addition, there was a positive association between those interested in reading the energy labelling and their knowledge of how to calculate daily caloric intake (*p*-value = 0.001).

### 3.5. The Observation of Energy Labelling and Its Impact on Participants’ Food Choices during the COVID-19 Pandemic

Only 17% of participants reported an issue using energy labelling during the COVID-19 pandemic, with 45% reporting no issues and 38% uncertain about their responses. The issues included energy labelling not being displayed next to all meals on the menu, energy labelling not displayed next to some meals on the menu, and no response from the restaurants when asking about energy labelling or inaccurate energy labelling.

During the COVID-19 pandemic, more than half (55%) of participants reported they had noticed energy labelling in the restaurant at their recent visit, with 21% not noticing it and 24% not sure if energy labelling was displayed. About 35% of participants who noticed the energy labelling said that their orders were not affected by the energy labelling, and 23% said they were not sure about their response at that time. However, 42% of the participants who noticed the energy labelling said it affected their order by changing the order (33%), ordering less food (23%), eating less fast food (36%), or choosing another restaurant (8%). Young (age 18–24) (*p* < 0.001), single (*p* = 0.022), and with bachelor’s degrees (*p* < 0.001) participants were significantly associated with noticing the menu labelling during restaurant visits.

Regarding the influence of providing energy information on food delivery applications during the COVID-19 pandemic, 39% of the participants reported that they had noticed energy labelling when using food delivery applications, 34% did not notice it, and 27% were unsure if energy labelling was available. About 39% of participants who noticed the energy labelling said it did not affect their order, and 28% were unsure about their responses. However, 33% of the participants who noticed the energy labelling said it affected their experience by changing the order (33%), eating less food (24%), eating less fast food (33%), or choosing another restaurant (9%). Young (age 18–24) (*p* < 0.001), single (*p* = 0.009), and students (*p* < 0.001) participants were significantly associated with noticing the menu labelling on food delivery applications.

## 4. Discussion

This observational population-based study aimed to learn more about how the COVID-19 pandemic impacted the public’s interest into using energy labelling on restaurant menus and food delivery applications across a large, young, and primarily healthy sample of Saudi adults. Our study is one of the first in Saudi Arabia to investigate the immediate impacts of COVID-19 on public interest and behaviour regarding caloric displays following home confinement. There were 1657 respondents, 81% female (*n* = 1343) and 69% aged 18–30 (*n* = 1141). The study found that during the COVID-19 pandemic, the participants’ interest in reading caloric information displayed on restaurant menus and meal delivery applications had not changed. Most participants claimed that they had little or no interest in energy labelling before or during the COVID-19 pandemic. However, despite their low interest, 75% agreed that energy labelling was helpful in improving individuals’ food choices during the COVID-19 pandemic.

This result contrasts with the findings of another major Saudi study that demonstrated considerable gains in closer public attention to nutritional panels and other kinds of food labelling when selecting foods [23]. This could be due to differences in the study population, as the majority of this study’s participants were young and healthy, with incomes of about 60% less than 2000 Saudi Riyal. In addition, this study survey was disseminated when the lockdown started to ease. This may have affected the participants’ interest in feeling free not to check the energy labelling.

Approximately 40% of the participants claimed that they had enough information to determine their daily caloric needs. This could be attributable to the unchanged BMI results, which showed that 46% of the participants were within the normal BMI range before and during the pandemic. This finding is consistent with the 47% of participants who reported their perceived weight as normal. However, during the COVID-19 pandemic, 33% of participants reported an increased weight. The weight gain could be because about 60% of the participants reported being inactive.

Since the pandemic, people prioritised their health to reduce infection risk by eating healthy [23]. This study found that 60% of the participants reported an adequate diet, with only 12% claiming that their diet was healthy before the pandemic. However, 32% of the participants reported improving their diet. Similar studies in the Netherlands, Poland, Spain, and Qatar reported that participants ate healthier meals during the lockdown [10,24,25,26]. Health consciousness—a person’s health concerns—explains this behaviour [27]. Good behaviours lead to healthy activities in health-conscious people. The pandemic raised health awareness [28]. This feature may be positive and essential in bringing about change, but it may not be enough, especially when changing eating habits [23]. This study found that 47% of the participants did not change their diet during the COVID-19 pandemic. The Transtheoretical Model’s phase hypothesis suggests that the coronavirus pandemic may have caused people to contemplate taking action to change their diet [29]. Thus, people may need assistance and educational plans to do so going forward.

During the COVID-19 pandemic curve, people ordering food from restaurants and home delivery applications increased, contributing to a higher daily caloric intake. Recent research in Saudi Arabia showed that 24–50% of diners used restaurant menus’ caloric information [5,6,8]. In this study, the percentage of restaurant visits (2–4 times/week) was the most frequent among participants (32%) before the COVID-19 pandemic. However, during the COVID-19 pandemic, visiting a restaurant once per week was the most frequent among 35% of the study participants. In addition, participants who reported not visiting a restaurant during the COVID-19 pandemic increased from 7% to 15%. The majority of participants had visited a restaurant 2–4 times/week prior to the pandemic but this decreased to once per week as a result of the lockdown. In this study, there was no change in use for meal delivery applications before or during the COVID-19 pandemic. Most participants (58%) reported being fairly active, and did not report any significant changes in their BMI during the COVID-19 pandemic. It has been reported that food ordering was significantly associated with weight gain after one month of lockdown among individuals in Malaysia [30]. In contrast, the participants in this study reduced their restaurant meals but did not increase their use of meal delivery services, thus maintaining the same BMI during the pandemic as prior to the pandemic.

During the COVID-19 pandemic, it became necessary to ensure that all restaurants disclosed the energy content of foods in both food menus and meal delivery applications to help people choose healthy food. In this study, 55% noticed energy labelling during their recent restaurant visits, with 42% saying that the energy information changed their meal selections. In addition, 40% of the participants noticed the energy information available on the meal delivery applications, and 33% claimed that the energy labelling changed their orders. For both restaurants and meal delivery applications, the changes in behaviour encouraged participants to change orders, eat less, reduce fast food, or choose a different restaurant.

People’s nutritional knowledge is strongly correlated with the type of diet they consume, making education on nutrition one of the many factors necessary to bring about behavioural change. Our findings indicate that less than half of the participants believed they could determine their daily caloric needs. In addition, we found a statistically significant correlation between individuals’ interest in energy-labelling information and their knowledge of caloric calculation. These findings are consistent with earlier Saudi [5] and international studies [31,32]. Studies in the West have suggested that educational and nutritional awareness are crucial before making a food choice, increasing people’s adherence to the nutritional facts label [33,34]. There are various models of behavioural change, including the Transtheoretical Model [35], the social cognitive theory [36], and the planned behavioural theory [37], that recognise the importance of nutritional-related knowledge, attitudes, and motives of individuals. These “volitional behaviour” models presume a logical connection between a person’s thoughts, feelings, intentions, and actions. According to Sapp [38], a “high threshold level of ‘how to’ and ‘awareness’ nutrition knowledge” is essential for individuals to decide on healthy choices for eating. On the contrary, erroneous assumptions and inadequate information could create irrational eating habits. Although some studies suggest that nutritional knowledge is irrelevant to eating behaviours, nutritional information is necessary for individuals who adopt a healthy diet and cannot be ignored.

The results of this study should be interpreted with caution due to some limitations. It should be noted that a cross-sectional study can only address relationships between variables; it cannot prove causality. Our study’s sociodemographic data may not reflect the proportional structure of the entire Saudi population because we used convenience sampling and social media platforms like Twitter and WhatsApp to disseminate the electronic questionnaire, which may have introduced some minor bias. Convenience sampling is the most efficient and economical way to collect data from a sizeable demographic group [39]. In addition, the online survey was a quick and cost-effective approach to collect self-reported data on participants’ knowledge, attention, and behaviour regarding caloric display following home confinement in Saudi Arabia. This was a valuable method for collecting a large number of responses when face-to-face interviews were difficult due to lockdowns. Furthermore, the perception questions did not evaluate whether people knew their energy requirements. However, the study’s descriptive findings offer a starting point for exploring how the public reacts to the COVID-19 lockdown regarding their interest in menu caloric labelling.

## 5. Conclusions

The inconsistent changes of food intake that are reported by individuals may not indicate healthy eating patterns. Still, the pandemic may have instilled a sense of personal responsibility for one’s health and boosted people’s nutritional interest among the examined Saudi adult population. In Saudi Arabia, the rate of restaurant delivery and takeout has increased, along with the prevalence of obesity and sedentary lifestyles. To encourage a healthy diet and reduce daily calories, it could be helpful to review energy-labelling practices to enhance their use in both normal and health emergency conditions. For those already at a higher risk of obesity, this is of paramount importance, especially during times of crisis and emergency. Education and guidance on energy labelling must be continued to help improve food choices.

## Figures and Tables

**Table 1:** Characteristics and backgrounds of participants (*n* = 1657).

Variables	*n*	%
**Age (y)**		
18–24	854	52
25–39	524	32
40–59	255	15
≥60	24	1
**Gender**		
Male	314	19
Female	1343	81
**Nationality**		
Saudi	1539	93
Non-Saudi	118	7
**Marital Status**		
Single	968	58
Married	574	35
Widower	15	1
Divorced	59	4
Preferred not to say	41	2
**Educational Level**		
High school or less	646	39
University level	877	53
Higher degrees	134	8
**Work Status**		
Student	743	45
Employed	510	31
Unemployed	241	15
Retired	163	10
**Income (SR) ^a^**		
No income	482	29
<2000 SR	476	29
2000–4000 SR	206	12
5000–7000 SR	136	8
8000–10000 SR	133	8
>10,000 SR	224	14
**Field of Study**		
Scientific	615	38
Literature	498	30
Medical	408	25
No specific field	114	7
**Region**		
Central region	499	30
Northern region	68	4
Southern region	53	3
Eastern region	184	11
Western region	853	51
**Diagnosed with COVID-19**		
Yes	236	14
No	1421	86
**Medical Diagnosis**		
Reported no diseases	1325.6	80
Cardiovascular diseases ^b^	83.1	5
Osteoporosis	57.1	3
Diabetes	72.1	4
Anemia	123.1	7
**Smoking**		
Yes	115	7
No	1497	90
Ex-smoker	45	3
**Physical Activity**		
Fairly inactive	960	58
Moderately active	538	32
Very active	159	10
**Knew How to Calculate Calories**		
Yes	671	40
No	652	39
Not sure	334	20
**Views About the Helpfulness of Energy Labelling During the COVID-19 Pandemic to Improve Food Choices**		
Agree	1241	75
Disagree	22	1
Neutral	235	14
Did not know	159	10

*n* (%) = Data presented as number and percentage; ^a^ SR = Saudi Riyal; ^b^ Included hypertension, hypercholesterolemia, and hypertriglyceridemia.

**Table 2:** Perceived weight status, body weight, and quality of diet before and during the COVID-19 pandemic (*n* = 1657).

		Answers	*n*	%
**Perceived Weight Status**	**Before COVID-19 Pandemic** (*n* = 1657)	Below normal	181	11
		Within the normal range	774	47
		Slightly above the normal weight	515	31
		Above the normal weight	123	7
		Not sure	64	4
	**During COVID-19 Pandemic** (*n* = 1657)	Yes, gained weight	554	33
		Yes, lost weight	379	23
		No, weight had not changed	528	32
		Not sure	196	12
Body Mass Index *	**Before COVID-19 Pandemic** (*n* = 1624)	Underweight	210	13
		Normal	741	46
		Overweight	410	25
		Obese	263	16
	**During COVID-19 Pandemic** (*n* = 1622)	Underweight	201	12
		Normal	761	47
		Overweight	378	23
		Obese	282	17
**Perceived Diet Quality**	**Before COVID-19 Pandemic** (*n* = 1657)	Unhealthy	420	25
		Adequate	990	60
		Healthy	194	12
		Did not know	53	3
	**During COVID-19 Pandemic** (*n* = 1657)	Yes, it improved it	523	32
		Yes, it made it worse	352	21
		No, it did not affect it	782	47

* Calculated based on self-reported weight and height.

**Table 3:** Interest in reading the energy labelling before and during the COVID-19 pandemic in restaurants and meal delivery applications (*n* = 1657).

Variables	Restaurant Visits						Food Delivery Applications					
	Before COVID-19			During COVID-19			Before COVID-19			During COVID-19		
	Extremely interested	Little interested	Not interested	Extremely interested	Little interested	Not interested	Extremely interested	Little interested	Not interested	Extremely interested	Little interested	Not interested
Total (%)	17	39	43	22	39	38	17	33	50	20	33	46
**Age (y)**												
18–24	152 (9)	295 (18)	407 (25)	211 (13)	284 (17)	359 (22)	151 (9)	252 (15)	451 (27)	188 (11)	252 (15)	414 (25)
25–39	93 (6)	233 (14)	198 (12)	103 (6)	245 (15)	176 (11)	81 (5)	207 (12)	236 (14)	101 (6)	205 (12)	218 (13)
40–59	40 (2)	115 (7)	100 (6)	52 (3)	112 (7)	91 (5)	39 (2)	88 (5)	128 (8)	45 (3)	92 (6)	118 (7)
≥60	4 (0)	8 (0)	12 (1)	6 (0)	7 (0)	11 (1)	4 (0)	7 (0)	13 (1)	4 (0)	6 (0)	14 (1)
*p*-value	0.003			<0.000			0.019			0.011		
**Gender**												
Male	62 (4)	114 (7)	138 (8)	69 (4)	124 (7)	121 (7)	58 (4)	98 (6)	158 (10)	63 (4)	103 (6)	148 (9)
Female	227 (14)	537 (32)	579 (35)	303 (18)	524 (32)	516 (31)	217 (13)	456 (28)	670 (40)	275 (17)	452 (27)	616 (37)
*p*-value	0.35			0.973			0.496			0.921		
**Marital Status**												
Single	181 (11)	350 (21)	437 (26)	242 (15)	346 (21)	380 (23)	182 (11)	303 (18)	483 (29)	215 (13)	302 (18)	451 (27)
Married	88 (5)	260 (16)	226 (14)	106 (6)	267 (16)	201 (12)	77 (5)	218 (13)	279 (17)	96 (6)	222 (13)	256 (15)
Widower	0 (0)	5 (0)	10 (1)	2 (0)	3 (0)	10 (1)	1 (0)	3 (0)	11 (1)	3 (0)	2 (0)	10 (1)
Divorced	10 (1)	24 (1)	25 (2)	13 (1)	19 (1)	27 (2)	10 (1)	17 (1)	32 (2)	13 (1)	18 (1)	28 (2)
Preferred not to say	10 (1)	12 (1)	19 (1)	9 (1)	13 (1)	19 (1)	5 (0)	13 (1)	23 (1)	11 (1)	11 (1)	19 (1)
*p*-value	0.011			0.001			0.04			0.034		
**Educational Level**												
High school or less	78 (5)	227 (14)	341 (21)	114 (7)	225 (14)	307 (19)	74 (4)	201 (12)	371 (22)	95 (6)	208 (13)	343 (21)
University level	174 (11)	368 (22)	335 (20)	218 (13)	369 (22)	290 (18)	170 (10)	309 (19)	398 (24)	208 (13)	307 (19)	362 (22)
Higher degrees	37 (2)	56 (3)	41 (2)	40 (2)	54 (3)	40 (2)	31 (2)	44 (3)	59 (4)	35 (2)	40 (2)	59 (4)
*p*-value	<0.000			<0.000			<0.000			<0.000		
**Work Status**												
Student	122 (7)	272 (16)	349 (21)	179 (11)	253 (15)	311 (19)	124 (7)	233 (14)	386 (23)	160 (10)	229 (14)	354 (21)
Employed	115 (7)	214 (13)	181 (11)	134 (8)	216 (13)	160 (10)	107 (6)	182 (11)	221 (13)	122 (7)	181 (11)	207 (12)
Unemployed	37 (2)	93 (6)	111 (7)	41 (2)	97 (6)	103 (6)	33 (2)	82 (5)	126 (8)	41 (2)	81 (5)	119 (7)
Retired	15 (1)	72 (4)	76 (5)	18 (1)	82 (5)	63 (4)	11 (1)	57 (3)	95 (6)	15 (1)	64 (4)	84 (5)
*p*-value	<0.000			< 0.000			< 0.000			< 0.000		

*n* (%) = Data presented as number and percentage. The *p*-values for chi-squared tests were calculated among age, gender, marital status, educational level, and work status.

## Data Availability

The data used to support the findings of this study are available from the corresponding author upon request.
